# ViMOP: a user-friendly and field-applicable pipeline for untargeted viral genome nanopore sequencing

**DOI:** 10.1093/bioinformatics/btaf687

**Published:** 2025-12-29

**Authors:** Nils Peter Petersen, Mia Le, Annick Renevey, Ehizojie Emua, Sarah Ryter, Giuditta Annibaldis, Jacob Camara, Sanaba Boumbaly, Cyril Erameh, Tanja Laske, Jan Baumbach, Philippe Lemey, Stephan Günther, Sophie Duraffour, Liana Eleni Kafetzopoulou

**Affiliations:** Bernhard Nocht Institute for Tropical Medicine (BNITM), 20359 Hamburg, Germany; German Center for Infection Research (DZIF), Partner Site Hamburg–Lübeck–Borstel–Riems, 20251 Hamburg, Germany; Bernhard Nocht Institute for Tropical Medicine (BNITM), 20359 Hamburg, Germany; German Center for Infection Research (DZIF), Partner Site Hamburg–Lübeck–Borstel–Riems, 20251 Hamburg, Germany; Institute for Computational Systems Biomedicine, University of Hamburg, 22761 Hamburg, Germany; Bernhard Nocht Institute for Tropical Medicine (BNITM), 20359 Hamburg, Germany; German Center for Infection Research (DZIF), Partner Site Hamburg–Lübeck–Borstel–Riems, 20251 Hamburg, Germany; Viral and Emergent Pathogens Control and Research, Irrua Specialist Teaching Hospital, 310115 Irrua, Edo State, Nigeria; Bernhard Nocht Institute for Tropical Medicine (BNITM), 20359 Hamburg, Germany; German Center for Infection Research (DZIF), Partner Site Hamburg–Lübeck–Borstel–Riems, 20251 Hamburg, Germany; Bernhard Nocht Institute for Tropical Medicine (BNITM), 20359 Hamburg, Germany; German Center for Infection Research (DZIF), Partner Site Hamburg–Lübeck–Borstel–Riems, 20251 Hamburg, Germany; Virology Research Center/Laboratory of Viral Hemorrhagic Fevers, 001 Conakry, Guinea; Virology Research Center/Laboratory of Viral Hemorrhagic Fevers, 001 Conakry, Guinea; Viral and Emergent Pathogens Control and Research, Irrua Specialist Teaching Hospital, 310115 Irrua, Edo State, Nigeria; Institute for Computational Systems Biomedicine, University of Hamburg, 22761 Hamburg, Germany; Leibniz Institute of Virology, Viral Systems Modeling, 20251 Hamburg, Germany; Institute for Computational Systems Biomedicine, University of Hamburg, 22761 Hamburg, Germany; Department of Mathematics and Computer Science, Computational Biomedicine Lab, University of Southern Denmark, 5230 Odense, Denmark; Department of Microbiology, Immunology and Transplantation, KU Leuven, Rega Institute, 3000 Leuven, Belgium; Bernhard Nocht Institute for Tropical Medicine (BNITM), 20359 Hamburg, Germany; German Center for Infection Research (DZIF), Partner Site Hamburg–Lübeck–Borstel–Riems, 20251 Hamburg, Germany; Bernhard Nocht Institute for Tropical Medicine (BNITM), 20359 Hamburg, Germany; German Center for Infection Research (DZIF), Partner Site Hamburg–Lübeck–Borstel–Riems, 20251 Hamburg, Germany; Bernhard Nocht Institute for Tropical Medicine (BNITM), 20359 Hamburg, Germany; German Center for Infection Research (DZIF), Partner Site Hamburg–Lübeck–Borstel–Riems, 20251 Hamburg, Germany; Department of Microbiology, Immunology and Transplantation, KU Leuven, Rega Institute, 3000 Leuven, Belgium

## Abstract

**Motivation:**

Untargeted, also known as metagenomic, nanopore sequencing is a powerful tool for virus genomic surveillance, particularly in resource-limited settings and when paired with the portability of the MinION device (Oxford Nanopore Technologies, ONT). However, a major bottleneck for global access is the absence of a user-friendly software capable of efficiently analyzing untargeted nanopore sequencing data to generate high-quality consensus genomes.

**Results:**

We share ViMOP, a pipeline built on our long-term experience in nanopore field sequencing. The pipeline emphasizes field user-friendliness, flexibility and versatility to analyze reads generated directly from human clinical samples. The software assembles *de novo* contigs, matches contigs to known viral references and uses them to assemble consensus genomes. Executed with a single Nextflow command or via the EPI2ME Desktop interface (ONT), results are summarized in an HTML report. ViMOP, through its user-centered design, lowers the barrier to high-quality virus genome reconstruction and advances capacity for genomic surveillance.

**Availability and implementation:**

ViMOP is freely available for non-commercial use (https://github.com/opr-group-bnitm/vimop and https://zenodo.org/records/17913089), along with the associated database (https://zenodo.org/records/17652512), the scripts used to generate it (https://zenodo.org/records/17632662) and benchmarking code (https://zenodo.org/records/17633185).

## 1 Introduction

Genomic surveillance has proven essential for identifying and responding to emerging viral disease outbreaks such as with mpox and COVID-19 (Attwood *et al.* 2022, Kinganda-Lusamaki *et al.* 2025). Rapid characterization of viral agents, alongside dense coverage of consensus genomes to monitor virus evolution, has substantially informed outbreak control efforts. This approach has facilitated the reconstruction of chains of transmissions, provided insights into the origin(s) of virus (re)-emergence, and enabled the monitoring of potential vaccine escape variants (Quick *et al.* 2016, Sissoko *et al.* 2017, Harvey *et al.* 2021, Keita *et al.* 2021, Kinganda-Lusamaki *et al.* 2021). Today, real-time genomic data sharing serves as a cornerstone for public health surveillance and evidence-based decision making.

Outbreaks often occur in endemic regions with limited resources (Kinganda-Lusamaki *et al.* 2021, Koundouno *et al.* 2022, Kinganda-Lusamaki *et al.* 2025). Global disparities persist in access to genomic surveillance infrastructure, and solutions to facilitate broad access and field applicability must be prioritized (Brito *et al.* 2022, Carter *et al.* 2022). Nanopore-based sequencing using the MinION device (Oxford nanopore technologies, ONT) offers a portable approach suited for such settings (Quick *et al.* 2016, Quick *et al.* 2017, Kafetzopoulou *et al.* 2019, Keita *et al.* 2021, de Vries *et al.* 2022). We previously demonstrated the feasibility of untargeted (metagenomic) nanopore sequencing and on-site analysis during outbreak emergencies in low- and middle-income countries (LMICs) (Kafetzopoulou *et al.* 2019, Keita *et al.* 2021, Koundouno *et al.* 2022).

Recurring challenges in on-site field data analysis include the highly demanding technical setup in environments with unreliable power supply and internet access, as well as the need of advanced bioinformatics expertise. Operating bioinformatics tools from the command line requires expertise that may not be available when trained personnel are scarce. Also, factors such as field-sample quality (e.g. lack of cold chain) and sample type (e.g. blood, swab or seminal fluid) may lead to large fractions of host-reads, short reads or uneven read coverage, challenges that analysis pipelines should be equipped to handle. Ultimately, generating high-quality consensus genomes is a requirement for further downstream molecular analysis, and sharing with public health authorities (Carter *et al.* 2022).

Few tools support automated analysis of untargeted viral nanopore data (Table 1, available as supplementary data at *Bioinformatics* online). None fully satisfied our requirements for offline use, simple setup and operation by non-experts. Vir-MinION and VirPipe support viral detection but do not automate the transition to reference-based assembly, instead relying on *de novo* reconstruction that is often fragmented for untargeted sequencing data (Mastriani *et al.* 2022, Kim *et al.* 2023). VirDetector is limited when host reads, segmented viral genomes, or co-infections are present (Kaiser *et al.* 2025). INSaFLU-TELEVIR is the only option with a graphical interface, yet still requires command-line installation, separates detection and assembly, and depends on a large local database (Santos *et al.* 2024).

We thus developed ViMOP (Virus Metagenomics for Outbreaks Pipeline), a user-friendly, automated pipeline for virus detection and reference-guided genome assembly of untargeted sequencing reads from human clinical or animal samples. Integration into ONT’s EPI2ME framework enables command-line-free operation, setup and updates. Alternatively, it runs with a single Nextflow command. Docker containers grant independence from the host operating system. ViMOP runs offline on a laptop (≥16 CPU cores, 30 GB RAM recommended) enabling flexible deployment.

## 2 Materials, methods, and results

### 2.1 Broad overview of the nanopore virus surveillance pipeline

**Figure 1.**
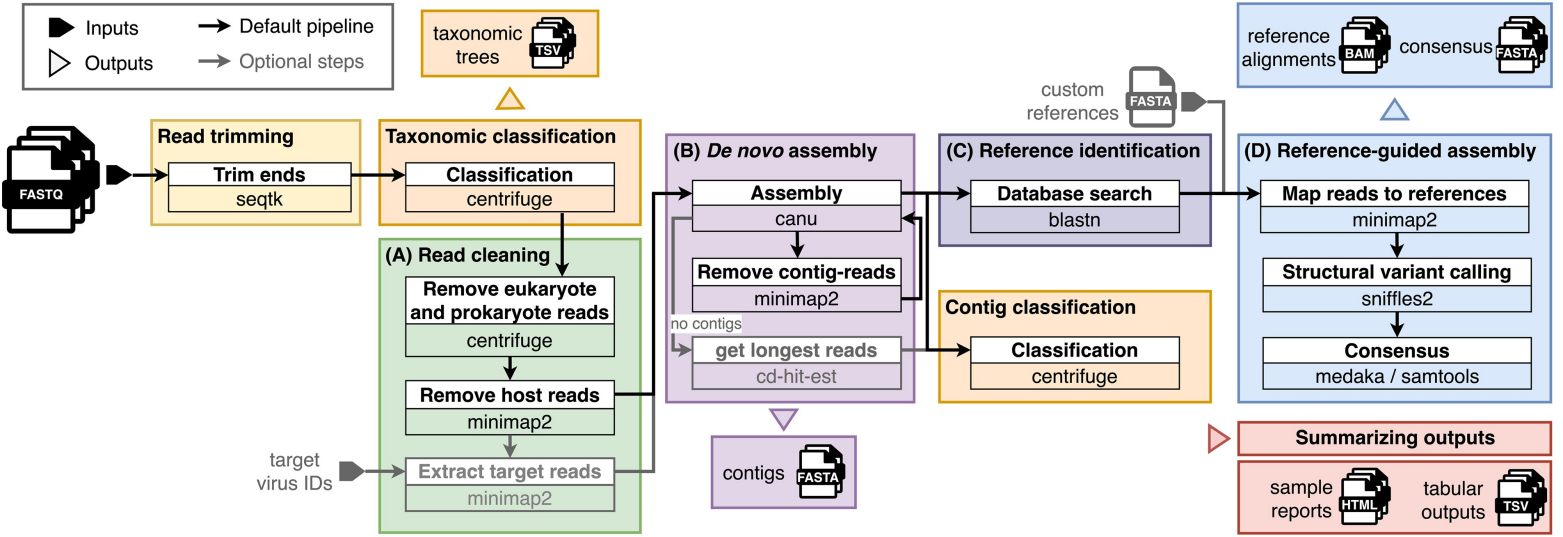
Schematic overview ViMOP. The workflow starts with cleaning and filtering the reads (A) which are then assembled into *de novo* contigs (B). Closely related virus genomes are identified (C) and used for a reference-guided assembly (D). Results are provided in an output directory with consensus sequences, alignments and TSV files and summarized in a comprehensive HTML report.

ViMOP detects known viral species and generates consensus genomes from basecalled nanopore reads (FASTQ) (Fig. 1; see Table 2, available as supplementary data at *Bioinformatics* online for tool choice rationales). Following separation of viral from non-viral reads, viral reads are assembled *de novo* into contigs. These are used to identify closely related reference genomes from a local database. Viral consensus sequences are subsequently generated via reference-guided assembly. All results, including consensus sequences and alignments, are output along with an HTML report.

### 2.2 Installation

ViMOP can be installed via EPI2ME Desktop by entering the GitHub repository URL. After installation, the backend database can be downloaded using the setup functionality. Alternatively, these steps can be done in a single Nextflow command.

### 2.3 Database

ViMOP uses a structured backend database and provides functionalities for automated download and updates. Users can create their own database. Our database is tailored to hosts and viral species relevant to our research. It contains (i) sequence files for background read removal, (ii) a virus genome set, and (iii) a Centrifuge index for read and contig classification. For background read removal, our database comprises the human genome and transcriptome, two rodent genomes and one mosquito genome, as well as a set of reagent-associated sequences including common bacterial laboratory strains and expression vectors (Tables 3 and 4, available as supplementary data at *Bioinformatics* online). The virus genome set is built based on all GenBank virus genomes (Benson *et al.* 2013). A curated subset of species relevant to our projects is maintained to ensure quality and consistency (Table 5, available as supplementary data at *Bioinformatics* online). Genomes with (a) >1% of unknown bases (N), (b) >98% sequence identity, or (c) <80% of a reference genome size, or not aligning to any reference genome (considered misassigned) were excluded. For SARS-CoV-2, a pre-selection from RVDB (Goodacre *et al.* 2018) was used. All remaining GenBank viral genomes are included without curation, and may require additional review upon detection. Groups comprising all genomes in our database for selected species or families were defined to filter for virus reads (Tables 5 and 6, available as supplementary data at *Bioinformatics* online). Our Centrifuge index combines our virus genome set with sequences (RefSeq) of human, mouse, bacterial, and archaeal genomes (O’Leary *et al.* 2016). Database updates and extensions are implemented as needed or upon user request. In low-connectivity settings, users may restrict an update to individual components.

### 2.4 Read trimming

seqtk (Li, https://github.com/lh3/seqtk) trims 3’ and 5’ ends to remove primer sequences. The number of trimmed-bases is user-configurable.

### 2.5 Taxonomic classification and read cleaning

Non-viral reads are removed in three steps. First, the reads are taxonomically classified using Centrifuge (Kim *et al.* 2016). Reads classified as non-viral are removed, and an overview of the microbial content of the sample is displayed in the ViMOP report. Second, for thorough removal of non-viral reads, the remaining reads are mapped with minimap2 (Li 2018) against one or more sets of host genome sequences and reagent-associated sequences. The resulting output is called the cleaned read set. Third, ViMOP filters for viral reads. This is optional. The user can apply one or multiple filters in parallel to specifically map the reads against one or more virus species or family. All reads are mapped against target viral genomes using minimap2. The reads mapping to a viral reference are called target filtered reads and one read set is created for each filter. For both the set of cleaned reads, and each set of target-filtered reads, *de novo* assembly and reference genome searches are performed.

### 2.6 *De novo* assembly

Reads are assembled into contigs using Canu (Koren *et al.* 2017). An iterative approach was implemented to account for uneven genome abundance (e.g. in a co-infection, one viral species might be less abundant than the other), increasing the likelihood for broad viral species detection. At each iteration, reads mapping against any generated contigs are removed. The remaining reads are further used for another Canu run. This is done until a pre-defined maximum number of iterations is reached, or until no contig is created or no reads are left. Assembled contigs are then used for reference identification. A special routine is integrated in ViMOP for Canu runs of target filtered read sets that did not yield any contigs. In this case, reads are clustered using CD-HIT (Fu *et al.* 2012) and then used as queries, analogous to contigs.

### 2.7 Reference identification

Contigs are matched to reference genomes from the backend database using BLAST (Altschul *et al.* 1990). For each contig, the top hit is selected using the default BLAST ranking with ties being resolved by the database’s internal ordering. Each unique hit is then used for reference-guided assembly. Users can manually add additional reference sequences in a FASTA file.

### 2.8 Reference-guided assembly

ViMOP assembles a consensus sequence for each reference identified. All reads are mapped to the reference sequence and large insertions and deletions are detected with Sniffles2 (Smolka *et al.* 2024). These are integrated into a first draft genome using BCFtools (Danecek *et al.* 2021). All reads are mapped to this draft, and the final consensus is built using either Medaka (ONT, https://github.com/nanoporetech/medaka) or Samtools (Danecek *et al.* 2021) to call single-nucleotide variants and small insertions and deletions. Per default, ViMOP uses Medaka if a model that fits the respective basecaller is available, and Samtools if not. In both cases, a minimum depth (default 20) is required to call bases. Samtools additionally requires a minimum proportion (70%) for a nucleotide to be called. Positions below the thresholds are masked with Ns. Samtools may alter the length of N-masked areas due to insertion or deletion artifacts from sparse reads. Therefore, a custom script replaces deletions that are not covered by the minimum depth of reads with Ns and removes insertions of Ns.

### 2.9 Contig classification

Contigs are classified using Centrifuge. This can help to identify contigs that were not assigned to a reference or are only partially aligned to the reference hit, e.g. if these are from bacterial origin. The Centrifuge classification is only used for reporting and not for the assembly of the reference target selection.

### 2.10 Output

ViMOP produces consensus sequences (FASTA), contigs (FASTA), alignment files (BAM), tables as tab-delimited files, and a separate HTML report for each sample. The user-friendly report, organized in three sections, provides information on the outcome of all the pipeline steps. The first section includes read distributions, read filtering statistics, and an overview of the Centrifuge read classification. The second section lists the viruses identified and statistics on genome recovery. The third section lists all *de novo* assembled contigs with the corresponding BLAST hits, and Centrifuge classification.

### 2.11 Benchmark

We compared ViMOP against INSaFLU-TELEVIR and VirDetector using simulated viral reads, mixed host–virus datasets, and real samples from the Sequence Read Archive (Supplementary Methods, Results, and Tables 7–12, available as supplementary data at *Bioinformatics* online). While VirDetector failed to automatically assemble many of the genomes, ViMOP and INSaFLU-TELEVIR show comparable results in most cases. Comparison on simulated data shows, that ViMOP is more robust when it comes to high mutation levels. Different to INSaFLU, ViMOP also recovers partial genomes below 50% completeness. On the downside, ViMOP has longer runtimes.

## 3 Conclusions and outlook

ViMOP simplifies untargeted nanopore sequencing data analysis. By integrating field-applicability, accessibility, maintainability and versatility, ViMOP enables broad usage for virus detection and genome assembly. The graphical interface, offline capability, and HTML reports make it particularly attractive for small teams focused on laboratory implementation. ViMOP integrates 8-years of experience of nanopore sequencing development in laboratories across Sub-Saharan Africa, and within the European Mobile Laboratory (https://www.emlab.eu). Integrated into our laboratory network, ViMOP will be continuously improved. ViMOP aims to strengthen global outbreak preparedness by supporting rapid, decentralized virus genomic surveillance where it is most urgently needed.

## Supplementary Material

btaf687_Supplementary_Data

## Data Availability

All software and resources associated with ViMOP are available via GitHub and Zenodo as cited in the Availability and Implementation section of the abstract. Benchmarking data consisted of publicly available SRA datasets and simulated reads. Simulated reads can be reproduced and SRA datasets downloaded using the benchmarking scripts provided with the benchmarking package.
