# Supplementary material for: ViMOP: a user-friendly and field-applicable pipeline for untargeted viral genome nanopore sequencing
Source: Bioinformatics. 2025 Dec 29;42(1):btaf687. doi: 10.1093/bioinformatics/btaf687 (PMC12809542; doi:10.1093/bioinformatics/btaf687)
Supplement: btaf687_Supplementary_Data [file btaf687_supplementary_data.pdf]

Supplementary Information

To

Title

ViMOP: A user-friendly and field-applicable pipeline for untargeted viral genome nanopore sequencing

Authors

Nils Peter Petersen [1,2,*,✉], Mia Le [1,2,3,*], Annick Renevey [1,2,], Emua Ehizojie [4], Sarah Ryter [1,2], Giuditta Annibaldis [1,2], Jacob Camara [5], Sanaba Boumbaly [5], Cyril Erameh [4], Tanja Laske [3,6], Jan Baumbach [3,7], Philippe Lemey [8], Stephan Günther [1,2], Sophie Duraffour [1,2,†], and Liana Eleni Kafetzopoulou [1,2,8,†]

* contributed equally

[†] contributed equally

[✉]corresponding author: Nils Peter Petersen, Bernhard Nocht Institute for Tropical Medicine (BNITM), Bernhard Nocht Straße 74, 220359, Hamburg, Germany; nils.petersen@bnitm.de, phone: +49 40 285380 641

Table of Contents

SUPPLEMENTARY METHODS **3**

PIPELINE EXECUTION AND PARAMETER CHOICES 3

ARTIFICIAL VIRUS GENOMES 3

READ SIMULATION 4

SUPPLEMENTARY RESULTS **5**

PREVIOUSLY PUBLISHED PIPELINES COMPARED TO VIMOP AND RATIONALE FOR SELECTION 5

SIMULATED VIRUS GENOMES 6

GENOME RECONSTRUCTION IN A SIMULATED HOST–VIRUS READ MIXTURE 7

SIMULATED VIRUS GENOMES IN CO-INFECTION AND MULTI SEGMENT MIXTURES 8

APPLICATION TO SRA DATA SETS 9

RUN TIME REPORT 10

SUPPLEMENTARY TABLES **11**

TABLE S1: PIPELINE COMPARISON 12

TABLE S2: TOOL CHOICES 13

TABLE S3: LIST OF HOST GENOMES AND REAGENT SEQUENCES 14

TABLE S4: SEQUENCES IN REAGENT SET 15

TABLE S5: LIST OF CURATED VIRUS DATA SETS 16

TABLE S6: LIST OF VIRUS TARGET FILTER FAMILIES 17

TABLE S7: BENCHMARKING CONSENSUS SEQUENCE QUALITY WITH SIMULATED VIRAL READS 18

TABLE S8: COMPOSITION OF BACKGROUND READS 19

TABLE S9: BENCHMARKING CONSENSUS SEQUENCE QUALITY WITH BACKGROUND READS 20

TABLE S10: SRA USE CASE DATA SETS 21

TABLE S11: GENOME RECONSTRUCTION FOR SRA USE CASES 22

TABLE S12: RUN TIMES 23

LITERATURE **24**

Supplementary Methods

Pipeline execution and parameter choices

For the comparative evaluation, ViMOP was benchmarked against INSaFLU-TELEVIR and VirDetector (see Supplementary Results for tool selection rationale). Both ViMOP and INSaFLU-TELEVIR trim 30 nucleotides from read ends by default to remove primers; because the simulated datasets did not contain primer sequences, this trimming parameter was set to 0 in both workflows. INSaFLU-TELEVIR applies a stricter minimum coverage threshold than ViMOP, therefore INSaFLU-TELEVIRs cutoff was adjusted to 20× to ensure comparability. VirDetector does not impose a coverage threshold. INSaFLU also includes a minimum genome recovery threshold. Consensus genomes below this threshold are not reported. We set this value to 50%, which is the smallest possible value.

As TELEVIR performs taxonomic detection on metagenomic data, it was first used to identify the best-scoring reference for each species. That reference was then selected or uploaded into the INSaFLU genome reconstruction module. VirDetector was run in its “hybrid assembly” mode, which combines de novo assembly (Canu), reference identification via BLAST, and subsequent reference-guided assembly. Since this approach most closely reflects ViMOP’s automated detection-plus-assembly strategy, only the hybrid mode was included in the comparison.

Apart from the adjustments described above, all pipelines were executed using default parameters.

Artificial virus genomes

We simulated artificial viral genomes to evaluate the robustness of downstream analyses against previously unobserved genomic variation. The objective was not to generate biologically realistic genomes, but to introduce mutations along the sequence in a way that reflects typical patterns of intra-species diversity, such as conserved regions, variable regions,

and higher substitution rates at third codon positions. Genomes from Lassa virus (both segments), dengue virus, and SARS-CoV-2 were aligned using MAFFT version 7.526 (Katoh and Standley, 2013) with default settings. For each multiple sequence alignment, a consensus sequence was determined and the number of genomes that differed from the consensus at each position was recorded. This count served as a positional weight, representing the empirical likelihood of mutation at that site. A template genome was then selected, and a predefined fraction of sites (e.g., 10%) was designated for mutation. Positions were sampled without replacement according to the variability weights, such that highly variable sites were more likely to be selected than conserved ones. At each chosen position, the original nucleotide was replaced by one of the three alternative bases selected at random.

Read simulation

To generate sequencing reads for method validation, we used PBSIM version 3.0.5 (Ono, et al., 2022) to simulate nanopore reads based on an empirical error and length profile. The simulation parameters were derived from read length distributions characteristic of SISPA-based metagenomic sequencing (as in (Kafetzopoulou, et al., 2019)) ensuring that the synthetic data reflected the fragmentation patterns and error structure observed in real field-generated datasets. Reads were simulated both from the artificial viral genomes described above and from background material consisting of human and bacterial reference genomes.

Supplementary Results

This section describes computational experiments designed to compare ViMOP with two recently published pipelines for untargeted viral analysis of nanopore sequencing data: INSaFLU-TELEVIR and VirDetector. The comparison was performed using three types of datasets:

1. Simulated viral reads from single genomes or genome segments
2. Simulated mixtures of viral and host reads
3. Real sequencing reads downloaded from the Sequence Read Archive Sequence Read Archive (SRA; NCBI, https://www.ncbi.nlm.nih.gov/sra)

Previously published pipelines compared to ViMOP and rationale for selection

We identified available pipelines for viral analysis of untargeted nanopore sequencing data and systematically compared their features to those of ViMOP (Table S1). Among the evaluated tools, INSaFLU-TELEVIR and VirDetector were the most comparable in terms of scope and workflow, and were therefore selected for direct benchmarking.

INSaFLU-TELEVIR separates virus detection and reference-based assembly into two independent workflows. The TELEVIR module first identifies viral species present in the sample, after which the user must manually select and upload reference genomes to the INSaFLU module to generate consensus sequences. In contrast, ViMOP performs viral detection, reference selection, and consensus reconstruction within a single automated workflow.

VirDetector identifies a reference genome by mapping a de novo assembled contig and then constructs a consensus sequence. However, its design restricts the analysis to a single, non-segmented dominant genome per dataset, which prevents the detection of segmented viruses and co-infections.

VirMinion and VirPipe both support virus detection and de novo assembly, but neither produces reference-based consensus genomes. We installed VirMinion, but it generated only short contigs that were not comparable in completeness to those produced by the other tools, and its detection results were distributed across multiple output files, making interpretation difficult. For these reasons, VirMinion was excluded from benchmarking. VirPipe, although provided as a Docker container, failed to execute successfully on our system and was therefore also excluded.

Simulated virus genomes

To evaluate pipeline performance under controlled conditions, we generated a set of simulated viral genomes for four species included in our curated reference dataset: SARS-CoV-2, Dengue virus, and the Lassa virus S and L segments. For each genome, artificial variants were produced by introducing mutations ranging from 0 % to 30 % in 5 % increments. Nanopore reads were then simulated using PBSIM at an average depth of 30×. As expected for long-read sequencing, coverage was uneven across the genome—particularly reduced at the termini—so full genome reconstruction was not anticipated. Although this dataset lacks metagenomic background and therefore does not reflect real sample complexity, it serves as a controlled benchmark to assess reference selection, genome recovery, and reconstruction accuracy, as well as to probe performance limits at increasing divergence from available database genomes.

For each simulated sample, we measured (i) genome recovery as the percentage of genome positions successfully reconstructed (non-N bases) and (ii) the sequence identity between the reconstructed genome and the known simulated ground truth. The full results are provided in Table S7.

Across all tools, increasing mutation loads resulted in decreasing genome recovery and reduced detectability of the correct reference, with all methods failing to identify any target at 30 % divergence.

VirDetector detected only SARS-CoV-2. Because it does not apply a strict coverage threshold during consensus generation, it reconstructs larger portions of genomes than the other tools at low divergence (≤ 15%). However, this comes at the cost of introducing errors already at 15% and 20% mutation rate divergence, earlier than ViMOP.

Up to 25 % divergence TELEVIR (like ViMOP) detected all targets with one exception. For the Lassa segments, ViMOP still detected the target in three cases at ≥ 20 % mutation, whereas TELEVIR failed. Using INSaFLU reference-based genome assembly, genome recovery and accuracy were generally lower than for ViMOP. The discrepancy widened as mutation levels increased. INSaFLU suppresses reporting of consensus sequences when genome recovery falls below 50 %. At 20 % mutation rate INSaFLU does not build a consensus for all four viral genomes. In contrast, ViMOP reconstructed all four genomes with a coverage substantially higher than 50 % (Lassa S: 69.96 %, all others >85 %).

Overall, ViMOP demonstrated the most robust performance across mutation levels, detecting more targets and reconstructing genomes with higher completeness and accuracy than either VirDetector or INSaFLU-TELEVIR, particularly as divergence from reference sequences increased.

Genome reconstruction in a simulated host–virus read mixture

To assess the performance of ViMOP in a realistic scenario, we simulated a mixed sequencing dataset consisting of 500,000 background reads of human and bacterial origin combined with varying amounts of viral reads (Table S8). SARS-CoV-2 was selected as the viral target because it was the only genome consistently detected by all three comparison pipelines when using only viral reads as described previously. A mutated SARS-CoV-2 genome (5 % divergence) was used to avoid bias toward perfect reference matching. To evaluate the effect of viral read abundance on detection and assembly, four mixtures were generated containing 1305 (all

available reads), 1000, 500, or 250 viral reads. The results for the different tools are shown in Table S9 set 1 to 4.

VirDetector's hybrid assembly workflow failed to identify a viral reference in all four mixtures, demonstrating that the absence of read filtering prevents its use on untargeted host-rich datasets.

TELEVIR consistently detected the SARS-CoV-2 signal at all read depths, whereas INSaFLU was only able to reconstruct partial genomes when coverage was sufficient: 95.50 % and 73.57 % reconstruction from 1305 and 1000 reads, respectively.

ViMOP detected the correct reference genome down to 500 reads, in which case 1.13 % of the genome could still be reconstructed. Given that this level of recovery is below the threshold required for meaningful downstream analysis, the result demonstrates that ViMOP detects reference genomes across the full range of practically relevant read depths.

Overall, these results show that both INSaFLU–TELEVIR and ViMOP reliably detect the viral reference sequence at low abundance and in mixtures.

Simulated virus genomes in co-infection and multi segment mixtures

As a test for the detection of multiple virus genomes (co-infection) and segments of a segmented virus in one sample, we mixed the reads of all four simulated virus genomes with the background reads. For Dengue virus and SARS-CoV-2, we used the 5 % mutation-rate datasets. To allow a direct comparison between the two tools, we selected the unmutated read sets for analysis, because INSaFLU was unable to generate consensus genomes for the mutated datasets.

Both ViMOP and INSaFLU successfully detected all four viral segments and reconstructed them to the same extent observed in the single-virus experiments (Table S9 set 5). ViMOP automatically identified the appropriate reference genomes and produced the final assemblies

without user intervention. In contrast, INSaFLU required manual initiation of a separate reference-based assembly for each genome.

Application to SRA data sets

To evaluate the performance of the pipelines on real-world sequencing data, we selected four publicly available SRA datasets representing untargeted nanopore sequencing runs containing viruses from ViMOP's curated reference set: Zika virus, West Nile virus, Dengue virus, and SARS-CoV-2 (Table S10). The Zika, West Nile, and Dengue datasets originated from cell-culture experiments and therefore contained a high proportion of viral reads, whereas the SARS-CoV-2 dataset was generated from a human nasopharyngeal swab and was dominated by bacterial reads, with only a small fraction of viral material.

To further assess robustness in the detection of co-infections and highly imbalanced viral abundances, we created an additional "mixed" dataset by combining all reads from the Dengue sample (SRR32419728), the dataset with the highest number of virus reads (>147,000), with those from the SARS-CoV-2 sample (SRR15356294), the dataset with the lowest number of virus reads (<500).

Across the individual (non-mixed) datasets, VirDetector was able to reconstruct a complete genome for Dengue and Zika virus. It failed to assemble West Nile Virus and SARS-CoV-2, regardless of the input composition. In the mixed dataset, VirDetector again reconstructed Dengue but did not recover SARS-CoV-2.

TELEVIR and ViMOP successfully detected the reference genomes for all four viruses in both the standalone and mixed datasets. Both tools also reported the presence of Dengue and SARS-CoV-2 in the mixed sample, demonstrating sensitivity to low-abundance co-infecting genomes. INSaFLU showed the same behavior observed in the simulated benchmarks: the pipeline only produced consensus genomes when >50 % of a viral reference was covered. As a result,

INSaFLU generated assemblies for Zika and Dengue (where it recovered a few bases more than ViMOP) but not for West Nile virus or SARS-CoV-2.

Run time report

In Table S12 the run time for each tool was measured for two of the previously described datasets where at least two tools identified the consensus sequences successfully: the Dengue virus use-case and the simulated SARS-CoV-2 run that included 500,000 human and bacterial reads. VirDetector and ViMOP report the wall clock run time at the end of each run. For INSaFLU-TELEVIR the run time was measured manually for each step (preprocessing, virus detection, reference-based assembly).

In regards of run time, with more an hour ViMOP on average is substantially slower than INSaFLU-TELEVIR and VirDetector. This is because of the iterative re-assembly procedure that executes canu multiple times. To reduce the run time a user could reduce the maximum number of re-assemblies. If a target is already known (e.g., due to preceding PCR testing), users can also speed up the analysis using the virus filters of ViMOP to extract viral reads before the assembly and switching off and switching off the assembly of non-target reads. This way, the pipeline still searches for well-fitting reference sequences for all genome segments, but will not detect co-infections anymore. Doing so reduced the run time of the Dengue virus sample from SRA from more than 40 minutes to 22 minutes and that of the simulated SARS-CoV-2 data set from almost 2 hours to approximately 1 hour.

Supplementary Tables

Table S1: Pipeline comparison

Features offered by existing nanopore virus metagenomics pipelines in comparison to ViMOP, with emphasis on capabilities relevant to genomic surveillance.

		Vir-MinION	VirPipe	VirDetector	INSaFLU-TELEVIR	ViMOP
Usability	Containerized for host system independence and reproducibility (e.g with Docker)	❌	✅	✅	✅	✅
	Automatic set up and updates without command line usage	❌	❌	❌	🟡 (only for online tool)	✅
	Graphical user-interface	❌	❌	❌	✅	✅
	Execution in command line	✅	✅	✅	🟡 (separate installation)	✅
	Database and pipeline size <100GB	✅	✅	✅	❌	✅
Analysis	Detection of known viruses	✅	✅	✅	✅	✅
	Host depletion and viral enrichment	❌	✅	❌	✅	✅
	Consensus genome assembly	❌	❌	✅	🟡 (manual step in different moule)	✅
	Automated consensus genome assembly of segmented viruses and multiple viruses (co-infection)	❌	❌	❌	❌	✅
	Virus detection and assembly in one workflow	❌	❌	✅	❌	✅
	Summary table with names of detected and assembled virus species	❌	❌	❌	✅	✅

Table S2: Tool choices

Tools performing key steps and the reasons for including them in ViMOP.

Tool	Function	Reasoning for tool choice
**BLAST**	Detect viral reference genomes	Fast and sensitive search
**Canu**	*de novo* assembly	Highly customizable and handles mid-length long reads (300–1500 nt), unlike tools that discard shorter reads (e.g. Flye)
**CD-HIT**	Reduce the number of contigs or reads to a representative set. Reads are clustered when a virus filter to enrich the target was given and no contigs were assembled. Contigs are clustered in the iterative re-assembly procedure.	Fast and lightweight read clustering. Established, supported and well tested
**Centrifuge**	Taxonomic classification of reads and contigs	Uses a compact index which includes bacteria that fits in RAM and is downloadable, even with limited bandwidth
**Medaka**	Small variant calling (SNPs/indels)	ONT-maintained variant caller, expected to stay compatible with future flow cell chemistries
**minimap2**	Map reads for filtering host reads, virus target enrichment and for reference-based assembly	Fast, reliable, established and well supported
**seqtk**	Trim bases from read ends	Simple and flexible trimming of arbitrary primer sequences
**Sniffles2**	Long insertion and deletion calling	Fast long-read SV caller, optimized for ONT data

Table S3: List of host genomes and reagent sequences

These sequence sets can be used to deplete host and reagent sequences from input reads. The column "key" holds the value used to activate the given filter in ViMOP. All genomes were downloaded from GenBank.

Sequences	Accession	Key	Default usage
Human genome	GCF_000001405.40	human_dna	yes
Human transcriptome	GCF_000001405.40	human_rna	yes
Mouse genome	GCF_000001635.27	mouse	no
Mastomys natalensis genome	GCA_021653895.1	mastomys	no
Aedes aegypti	GCF_002204515.2	aedes_aegypti	no
Reagent set	see Table S3	reagent	yes

Table S4: Sequences in reagent set

Reagent-associated sequences included in the default ViMOP database for removal of non-viral reads. Part of this dataset was provided by David O'Connor (Department of Pathology and Laboratory Medicine), whom we gratefully acknowledge.

**Organism/Vector**	**Accession**
Penicillium chrysogenum	GCF_028827035
Ectopseudomonas mendocina	GCF_000733715
Bacteriophage lambda	NC_001416
Escherichia phage phiX174	NC_001422
Escherichia coli K-12	AP001918
Escherichia coli DH1	AP012030
Stutzerimonas stutzeri A1501	CP000304
Pseudomonas protegens Pf-5	CP000076
Pseudomonas fluorescens Pf0-1	CP000094
Pseudomonas fluorescens SBW25	OV986001
Pseudomonas fluorescens SBW25 plasmid pQBR103	AM235768
Expression vector pACT3	U51556
Expression vector pDawn	JN579121
Gateway binary vector R4pGWB759	AB608329
Cloning vector pT3TS-Cre	HQ335171
Cloning vector pHUE	AY751539
Cloning vector pTL61T	M29896

Table S5: List of curated virus data sets

For a list of virus taxa dedicated reference data sets were assembled via filtering to a list of quality constraints. The genomes were downloaded from NCBI virus with the exception of the "*severe acute respiratory syndrome coronavirus* 2" genomes downloaded from RVDB. Each of these datasets is also available as a virus target filter to limit the set of reads for the assembly to those that map to any reference identified in the dataset.

**Virus**	**Abbreviation**	**TaxID**
Emesvirus zinderi	MS2	329852
Lentivirus humimdef1	HIV1	3418650
Lentivirus humimdef2	HIV2	3418651
Mammarenavirus choriomeningitidis	LCMV	305230
Mammarenavirus juninense	JUNV	2169991
Mammarenavirus lassense	LASV	3052310
Orthoebolavirus	EBOV	3044781
Orthoflavivirus denguei	DENV	3052464
Orthoflavivirus zikaense	ZIKA	3048459
Orthomarburgvirus	MARV	3044783
Orthonairovirus hazaraense	HAZV	3052519
Severe acute respiratory syndrome coronavirus 2	COVID	2697049
West nile virus	WNV	3048448
Yellow fever virus	YFV	3046277

Table S6: List of virus target filter families

In addition to the filters for the curated data sets (**Table S3**) these sets of virus family genomes can be used to filter the reads. All genomes found for the given TaxID are downloaded from NCBI virus and added to the filter.

Virus family	Abbreviation	TaxID
Arenaviridae	ARENA	11617
Filoviridae	FILO	11266
Hantaviridae	HANTA	1980413
Nairoviridae	NAIRO	1980415
All genomes in virus reference database	ALL	10239

Table S7: Benchmarking consensus sequence quality with simulated viral reads

28 simulated datasets were generated with reference sequences containing point mutations at increasing rates. For each metric the best-performing value is highlighted in bold. VirDetector failed to identify Dengue or Lassa virus in all samples (marked “no hit”). In several cases INSaFLU-TELEVIR identified the correct reference, but did not generate a consensus sequence (marked “no consensus”), due to low genome recovery (<50%). For SARS-CoV-2, VirDetector generally recovered the highest percentage of bases from the mutated reference, but this came at the cost of lower sequence identity.

**reference virus (Accession)**	**Tool**	**Genome recovery [%]**			**Sequence identity (no Ns) [%]**		
	**mutation rate [%]**	**INSaFLU-TELEVIR**	**ViMOP**	**VirDetector**	**INSaFLU-TELEVIR**	**ViMOP**	**VirDetector**
SARS-CoV-2 (OX637002.1)	0	95.77	96.02	**99.85**	**100.00**	**100.00**	**100.00**
	5	95.5	96.01	**99.83**	99.99	**100.00**	**100.00**
	10	94.98	96.01	**99.24**	99.96	**100.00**	**100.00**
	15	63.68	95.57	**99.14**	99.87	**100.00**	99.99
	20	no consensus	**89.68**	89.39	no consensus	**100.00**	99.86
	25	no consensus	**47.79**	45.81	no consensus	99.15	**99.20**
	30	no hit	no hit	no hit	no hit	no hit	no hit
DENGUE 2 (NC_001474.2)	0	89.25	**89.45**	no hit	**100.00**	**100.00**	no hit
	5	89.18	**89.43**	no hit	**100.00**	**100.00**	no hit
	10	89	**89.24**	no hit	**100.00**	**100.00**	no hit
	15	65.27	**89.21**	no hit	99.86	**100.00**	no hit
	20	no consensus	**87.03**	no hit	no consensus	**100.00**	no hit
	25	no consensus	**17.66**	no hit	no consensus	**100.00**	no hit
	30	no hit	no hit	no hit	no hit	no hit	no hit
LASSA L segment (MG812630.1)	0	69.82	**86.77**	no hit	99.90	**100.00**	no hit
	5	no consensus	**86.77**	no hit	no consensus	**100.00**	no hit
	10	no consensus	**86.77**	no hit	no consensus	**100.00**	no hit
	15	no consensus	**86.59**	no hit	no consensus	**100.00**	no hit
	20	no hit	**85.81**	no hit	no hit	**99.98**	no hit
	25	no hit	**39.67**	no hit	no hit	**100.00**	no hit
	30	no hit	no hit	no hit	no hit	no hit	no hit
LASSA S segment (MG812631.1)	0	66.73	**79.92**	no hit	99.96	**100.00**	no hit
	5	no consensus	**79.92**	no hit	no consensus	**100.00**	no hit
	10	no consensus	**79.92**	no hit	no consensus	**100.00**	no hit
	15	no consensus	**79.34**	no hit	no consensus	**100.00**	no hit
	20	no hit	**70.14**	no hit	no hit	**99.96**	no hit
	25	no hit	no hit	no hit	no hit	no hit	no hit
	30	no hit	no hit	no hit	no hit	no hit	no hit

Table S8: Composition of background reads

Bacterial and the human reference genome were used to simulate a synthetic background dataset. For each organism, the corresponding RefSeq accession, absolute number of reads, and relative fraction of total reads are provided here.

Number of reads		Organism name	Accession
**Fraction**	**Absolute**		
**0.01**	5000	*Streptococcus mitis* SK637	GCF_000722765.2
**0.01**	5000	*Corynebacterium pseudodiphtheriticum* ACRQT	GCF_022345865.1
**0.01**	5000	*Capnocytophaga sputigena*	GCF_002302415.1
**0.02**	10000	*Moraxella catarrhalis*	GCF_002080125.1
**0.05**	25000	*Streptococcus pyogenes SF370* (M1 GAS)	GCF_000006785.2
**0.05**	25000	*Dolosigranulum pigrum* KPL3250	GCF_017655665.1
**0.05**	25000	*Staphylococcus aureus* NCTC8325	GCF_000013425.1
**0.05**	25000	*Pseudomonas putida* KT2440	GCF_000007565.2
**0.75**	375000	*Homo sapiens* (GRCh38.p14)	GCF_000001405.40

Table S9: Benchmarking consensus sequence quality with background reads

Simulated viral reads from Table S9 were combined with 500,000 simulated human and bacterial reads (see Table S8) to benchmark the capabilities of VirDetector, INSaFLU-TELEVIR and ViMOP with samples that contain large quantities of human and bacterial reads next to the viral reads. Data set 2-4 simulate cases with decreasing number of SARS-CoV-2 reads to benchmark the abilities to detect virus sequences even at very low virus concentrations. Data set 5 simulates a case of a co-infection of multiple viruses and segments. While the performance of INSaFLU-TELEVIR and ViMOP did not change compared to table S9, VirDetector failed to correctly identify any virus in the simulated samples and therefore is not listed in this table.

			**viral reads (% of whole dataset)**	**Genome recovery [%]**		**Sequence identity (no Ns) [%]**	
**dataset**	**reference virus**	**mutation rate [%]**		**INSaFLU-TELEVIR**	**ViMOP**	**INSaFLU-TELEVIR**	**ViMOP**
1	SARS-CoV-2	5	1305 (0.26%)	95.5	**96.01**	99.99	**100.00**
2	SARS-CoV-2	5	1000 (0.20%)	73.57	**76.84**	99.98	**100.00**
3	SARS-CoV-2	5	500 (0.10%)	no consensus	**1.13**	no consensus	**100.00**
4	SARS-CoV-2	5	250 (0.05%)	no consensus	no hit	no consensus	no hit
5	SARS-CoV-2	5	1305 (0.26%)	95.5	**96.01**	99.99	**100**
	DENGUE	5	484 (0.10%)	89.18	**89.44**	**100.00**	**100**
	LASSA L	0	324 (0.06%)	69.82	**86.77**	99.90	**100**
	LASSA S	0	151 (0.03%)	66.73	**79.92**	99.96	**100**

Table S10: SRA use case data sets

Data set	Virus species	Sample type	Submitted by	Publication date
SRR15356294	SARS-CoV-2	Human nasopharyngeal swab	University of British Columbia	2021-09-17
ERR14941561	West Nile virus	lab stock	University of Antwerp	2025-05-07
SRR31174237	Zika virus	lab stock	Wisconsin National Primate Research Center	2024-10-30
SRR32419728	Dengue virus	human serum-derived cultured isolate	Mahidol University	2025-03-07

Table S11: Genome reconstruction for SRA use cases

Number of nucleotides called was compared between INSaFLU-TELEVIR, ViMOP.

		**Positions called**		
	**Tool**	**INSaFLU-TELEVIR**	**ViMOP**	**VirDetector**
**Dataset**	**Target virus**			
SRR31174237	Zika virus	**10738**	10729	10683
ERR14941561	West Nile virus	no consensus	**2878**	no hit
SRR15356294	SARS-COV-2	no consensus	**11**	no hit
SRR32419728	Dengue virus	10675	10649	**10697**
SRR15356294<br>+ SRR32419728	SARS-COV-2<br>Dengue virus	no consensus<br>10675	**11**<br>10649	no hit<br>**10697**

Table S12: Run times

Run times were recorded for INSaFLU-TELEVIR, VirDetector using the SRA-Dengue use case (Table S10) and a simulated SARS-CoV-2 dataset with human and bacterial background reads (Table S9 set 1). Tools were executed on an 13th Gen Intel Core i9-13950HX × 32 laptop with 32 GB RAM and Ubuntu 20.04.4 LTS installed. VirDetector failed to detect SARS-CoV-2 so the corresponding run time is greyed out.

Dataset (number of reads)	Tool	Wall clock run time
SRR32419728<br>Dengue virus from SRA<br>(297971)	INSaFLU-TELEVIR	16m 45s
	ViMOP	44m 38s
	ViMOP with DENV filter	22m 45s
	VirDetector	1m 58s
Simulated<br>SARS-CoV-2 + Background<br>(501305)	INSaFLU-TELEVIR	20m 58s
	ViMOP	1h 55m 21s
	ViMOP with COVID filter	1h 04m 17s
	VirDetector	1m 16s (no hit)

Literature

Kafetzopoulou, L.E., *et al.* Metagenomic sequencing at the epicenter of the Nigeria 2018 Lassa fever outbreak. *Science* 2019;363(6422):74-77.

Katoh, K. and Standley, D.M. MAFFT multiple sequence alignment software version 7: improvements in performance and usability. *Mol Biol Evol* 2013;30(4):772-780.

Ono, Y., Hamada, M. and Asai, K. PBSIM3: a simulator for all types of PacBio and ONT long reads. *NAR Genom Bioinform* 2022;4(4):lqac092.
